# The Copper Homeostasis Transcription Factor CopR Is Involved in H_2_O_2_ Stress in *Lactobacillus plantarum* CAUH2

**DOI:** 10.3389/fmicb.2017.02015

**Published:** 2017-10-17

**Authors:** Yang Yang, Jia Yin, Jie Liu, Qi Xu, Tian Lan, Fazheng Ren, Yanling Hao

**Affiliations:** ^1^The Innovation Centre of Food Nutrition and Human Health (Beijing), College of Food Science and Nutritional Engineering, China Agricultural University, Beijing, China; ^2^Key Laboratory of Functional Dairy, Ministry of Education and Beijing Municipality, Beijing, China

**Keywords:** *Lactobacillus plantarum* CAUH2, H_2_O_2_ stress, CopR, Cu^2+^-exporting ATPase, copper homeostasis

## Abstract

Transcriptional factors (TFs) play important roles in the responses to oxidative, acid, and other environmental stresses in Gram-positive bacteria, but the regulatory mechanism of TFs involved in oxidative stress remains unknown in lactic acid bacteria. In the present work, homologous overexpression strains with 43 TFs were constructed in the *Lactobacillus plantarum* CAUH2 parent strain. The strain overexpressing CopR displayed the highest sensitivity and a 110-fold decrease in survival rate under H_2_O_2_ challenge. The importance of CopR in the response to H_2_O_2_ stress was further confirmed by a 10.8-fold increase in the survival of a *copR* insertion mutant. *In silico* analysis of the genes flanking *copR* revealed putative CopR-binding “*cop* box” sequences in the promoter region of the adjacent gene *copB* encoding a Cu^2+^-exporting ATPase. Electrophoretic mobility shift assay (EMSA) analysis demonstrated the specific binding of CopR with *copB in vitro*, suggesting *copB* is a target gene of CopR in *L. plantarum*. The role of CopB involved in oxidative stress was verified by the significantly decreased survival in the *copB* mutant. Furthermore, a growth defect in copper-containing medium demonstrated that CopB functions as an export ATPase for copper ions. Furthermore, EMSAs revealed that CopR functions as a regulator that negatively regulates *copB* gene and Cu^2+^ serves as inducer of CopR to activate the expression of CopB in response to H_2_O_2_ stress in *L. plantarum* CAUH2. Our findings indicated that CopR plays an important role in enhancing oxidative resistance by regulating *copB* to modulate copper homeostasis.

## Introduction

*Lactobacillus plantarum* is an aerotolerant Gram-positive bacteria belonging to the facultatively heterofermentative group of *lactobacilli* (Kleerebezem et al., 2003). Among lactic acid bacteria (LAB), this species has one of the largest genomes and a particularly high proportion of regulatory genes, allowing it to survive in a variety of different environmental niches. Consequently, *L. plantarum* is widely used as a starter culture in fermented food and feed production, such as dairy, meat, and many vegetables and plants (Leroy and De Vuyst, 2004). Moreover, some *L. plantarum* strains have been declared to have health-promoting effects, such as increasing iron absorption and enhancing immunity, and thus can also function as probiotic active ingredients in fermented foods (Hoppe et al., 2015; Hulst et al., 2015). However, *L. plantarum* suffers from unavoidable oxidative stress caused by reactive oxygen species (ROS) during manufacturing. ROS, including the superoxide anion (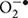), hydrogen peroxide (H_2_O_2_), the hydroxyl radical (HO^•^), and organic hydroperoxides (ROOH), can cause significant damage to various cellular structures such as DNA, proteins, and cell membranes (Imlay and Linn, 1988; Storz et al., 1990; Farr and Kogoma, 1991; Atack and Kelly, 2009). Therefore, *L. plantarum* has developed a variety of adaptive mechanism to cope with oxidative stress.

Bacterial cells respond to oxidative stress by inducing the expression of anti-oxidant enzymes. These mainly include superoxide dismutase (SOD), catalase, peroxidase, and glutathione peroxidase (Harris, 1992). However, *L. plantarum* lacks a SOD enzyme, and instead this bacterium accumulates a high intracellular concentration (20-30 mM) of manganese to scavenge superoxide and converts it to H_2_O_2_ during fermentative and aerobic growth (Archibald and Fridovich, 1981). In addition, the mannose phosphotransferase system (PTS) can enhance oxidative tolerance by transporting glucose to generate more ATP in *L. plantarum* WCFS1 (Stevens et al., 2010). Moreover, overexpression of the *trxB1* gene-encoding thioredoxin reductase in *L. plantarum* WCFS1 can also enhance H_2_O_2_ tolerance by inducing a group of 16 gene transcripts involved in purine biosynthesis, cell wall biosynthesis, energy metabolism, cellular envelope biosynthesis, and amino acid metabolism (Serrano et al., 2007).

In LAB, a few transcriptional factors (TFs) have been identified as regulators to modulate gene expression in various physiological processes. In *L. reuteri*, the PocR-like TF controls the expression of its neighboring loci that harbors the genes for glycerol utilization and vitamin B_12_ synthesis (Santos et al., 2011). A TerR-like TF (*lp_1153*) functions as a repressor of the *tarIJKL* locus involved in teichoic acid synthesis in *L. plantarum* WCFS1 (Tomita et al., 2015). In addition, TF Ldb0677 has been identified to involve in acid tolerance, which was predicted to regulate 22 target genes by bacterial one-hybrid method in *L. bulgaricus* CAUH1 (Zhai et al., 2014). Furthermore, the copper homeostasis system controlled by the CopY-type repressor has been extensively studied in *Enterococcus hirae* and *Lactococcus lactis* (Solioz et al., 2010). The core element of this system in *E. hirae* is the *cop* operon encoding four proteins: the repressor CopY, copper chaperone CopZ, copper import ATPases CopA, and copper export ATPases CopB. Transcription of this operon is controlled by CopY through binding to the “*cop box*” in the promoter region. However, the corresponding core element in *L. lactis* contained a *copRZA* operon and a monocistronic *copB*, which were both controlled by CopR. CopA was identified as a copper-exporting ATPase while function of CopB is unclear (Solioz et al., 2011). In addition, another CopR regulon *lctO* encoding an NAD-independent lactate oxidase plays an antioxidant role by attenuating the formation of reactive oxygen radicals in *L. lactis* (Barré et al., 2007).

*Lactobacillus plantarum* CAUH2 was isolated from Szechuan pickle, a traditionally fermented vegetable product from China. Whole-genome sequencing indicated that CAUH2 contained about 8.5% regulatory genes (GenBank accession no. CPO15126–CPO15129). Furthermore, 45 isogenous genes of TF with known functions were identified in the CAUH2 genome. However, the roles of these TFs in H_2_O_2_ resistance remain unknown. In the present study, homologous overexpression of 43 TF genes in *L. plantarum* CAUH2 was performed to identify potential involvement in H_2_O_2_-induced oxidative stress. Genetic and physical evidence revealed that the TF CopR plays an important role in enhancing oxidative resistance by negatively regulating *copB* to modulate copper homeostasis. To our knowledge, this is the first report of a TF involved in protection against oxidative stress in *L. plantarum*.

## Materials and Methods

### Bacterial Strains, Plasmids, and Growth Conditions

Bacterial strains and plasmids used in this study are listed in **Table 1**. *L. plantarum* CAUH2 cultures were incubated at 37°C in De Man Rogosa Sharp (MRS) medium, and *L. lactis* NZ9000 was grown at 30°C in GM17 (M17 broth supplemented with 0.5% w/v D-glucose). *Escherichia coli* cells were propagated aerobically at 37°C in Luria Bertani (LB) broth. When required, media were supplemented with the relevant antibiotics at the following concentrations: 25 μg mL^-1^ kanamycin, 25 μg mL^-1^ erythromycin for *E. coli*, 10 μg mL^-1^ chloramphenicol for *L. lactis*, 20 μg mL^-1^ chloramphenicol, and 10 μg mL^-1^ erythromycin for *L. plantarum*.

**Table 1:** Bacterial strains and plasmids used in this study.

Strain or plasmid	Relevant phenotype or genotype^a^	Source or reference
**Bacterial strains**		
*E. coli* DH5α	F^-^, φ80d*lacZ* Δ M15, Δ (*lacZYA–argF*)U169, *deoR*, *recA1*, *endA1*, *hsdR17*(r_K_^-^, m_K_^+^), *phoA*, *supE44*, λ^-^, *thi-1*, *gyrA96*, *relA1*	ΔTakara
*L. lactis* NZ9000	*L. lactis* MG1363 *pepN::nisRK*	deRuyter et al., 1996
*L. plantarum* CAUH2	Wild-type strain, isolated from koumiss, Catalase positive, glutathione peroxidase positive	Laboratory stock
**Plasmids**		
pSlpA8148	pNZ8148 derivative carrying constitutive promoter SlpA instead of PnisA, Cm _r_	Laboratory stock
pUC19	Kan_r_, pSC101 origin of replication	Vieira and Messing, 1982
pUC19EM	Suicide plasmid carried a Em_r_ cassette, derivative of pUC19	This work
pUC19EMcopR	pUC19EM derivative with a 316 bp copR fragment	This work
pNZCopR	pNZ8148 derivative containing *CopR*	This work

^a^*Kan^*r*^, kanamycin resistance; Em^*r*^, erythromycin resistance; and Cm^*r*^, chloramphenicol resistance*.

### DNA Manipulation Techniques

General molecular techniques including DNA electrophoresis analysis, recovery, and storage were performed using standard protocols (Sambrook et al., 1989). Miniprep plasmid isolation from both *E. coli* and *L. plantarum* was performed using the Plasmid Mini Kit I according to the manufacturer’s instructions (OMEGA Bio-tek Inc., Doraville, GA, United States). Genomic DNA from *lactobacilli* was prepared using the genomic DNA Extraction Kit (Tiangen, Beijing, China). Lysis of bacterial cells was carried out by adding lysozyme to TES buffer (50 mM Tris–HCl, 1 mM EDTA, 25% sucrose; pH 8.0) to a final concentration of 30 mg mL^-1^, and mixtures were incubated at 37°C for 1 h (Vandeguchte et al., 1989). Plasmids were introduced into *E. coli* DH5α using standard heat shock transformation, and electroporation was used for plasmid transfer into *L. plantarum* as described previously (Thompson and Collins, 1996). PCR products were amplified using *Ex Taq* polymerase (Takara, Dalian, China). DNA digestions with restriction endonucleases including *Nco*I, *Hin*dIII, *Kpn*I, *Pst*I, *Xba*I, and *Eco*RI were performed according to the supplier’s instructions (Takara, Dalian, China). DNA ligation was performed using the T_4_ DNA Ligation Kit (Thermo Fisher Scientific, Beijing, China) according to the manufacturer’s instructions. All primers and probes used in this study were synthesized by Sangon Biotech (Beijing, China). DNA sequencing was performed by Sangon Biotech and the results were further analyzed with the DNAMAN software package.

### Construction of Recombinant *L. plantarum* Strains with Putative Transcription Factor

Using the Collection of Manually Curated Inferences of Regulons in Prokaryotic Genomes database^1^, 57 isogenous TF genes with known functions were identified in the genome of *L. plantarum* WCFS1. Comparative genomics analysis showed that only 45 of these were present in the genome of *L. plantarum* CAUH2. These predicted TF genes were amplified using specific primers (listed in Supplementary Table S1) and inserted into the corresponding sites of pSlpA8148, resulting in a series of pSlpA8148-derived plasmids (designated as pSlpA-TF01 to pSlpA-TF43). The recombinant plasmids were sequenced and transformed into *L. plantarum* CAUH2 to generate corresponding recombinant strains CAUH2-TF01 to CAUH2-TF43. Meanwhile, a control strain (CAUH2-pSlpA8148) was constructed by introducing the empty pSlp8148 vector into *L. plantarum* CAUH2. To further investigate whether the TFs were successfully expressed, 5 of the 43 strains were randomly selected and SDS–PAGE analysis of cultures was performed.

### Response to H_2_O_2_ Stress

In order to determine the tolerance to H_2_O_2_ stress in the 43 *L. plantarum* recombinant strains, overnight cultures were inoculated into 10 mL of fresh MRS medium containing chloramphenicol. When cells reached an OD_600_ of 0.8, 1 mL of each culture was collected and centrifuged at 6000 × *g* for 2 min, then resuspended in the same volume of fresh MRS medium supplemented with 5 mM H_2_O_2_. After incubation for 30 min at 37°C, the number of colony-forming units per milliliter (CFU/mL) was determined by plating 10-fold serial dilutions on MRS agar containing chloramphenicol and incubating for 16 h at 37°C. Survival rates were calculated by dividing the CFU/mL values after H_2_O_2_ challenge by the value obtained immediately after resuspension. All results were obtained by at least three independent experiments and each biological replicate was performed in replicates.

### Insertional Inactivation of the *copR* Gene

To study the role of CopR in oxidative stress, a *copR* mutant of CAUH2 was constructed using the suicide plasmid pUC19EM. The EM_r_ cassette was amplified from the plasmid pGM36e using primers EM-F and EM-R (Supplementary Table S2) and inserted into the *Pst*I and *Hin*dIII sites of pUC19, resulting in the suicide plasmid pUC19EM. A 316 bp internal region of the *copR* gene was chosen as a homologous sequence and amplified using the primer pair copRHA-F and copRHA-R with flanking *Xba*I and *EcoR*I sites, respectively. The digested PCR product was ligated with the corresponding restriction sites of pUC19EM, and the recombinant plasmid, designated pUC19EMcopR, was introduced into *L. plantarum* by electroporation. Recombinants were selected for growth on MRS medium containing erythromycin. The inability to replicate plasmid pUC19EMcopR resulted in its integration into the *copR* gene region of the CAUH2 genome under erythromycin selection pressure. The resulting mutant was designated CAUH2ΔcopR. PCR was performed with forward primer EMT-F and reverse primer copRT-R to confirm integration of pUC19EMcopR at the correct genome locus. The primer EMT-F was designed according to the DNA sequence of erythromycin resistance genes (GenBank accession no. KM017875.1) and the primer copRT-R was designed according to the DNA sequence of CopR (A1F92_RS13920). Meanwhile, insertional inactivation of the *melA* gene was carried out by the same procedure to construct a control strain (CAUH2ΔmelA). The forward primer melAHA-F and the reverse primer melAHA-R were used to amplify the mutagenic fragments of *melA*. To confirm the integration at the correct locus, PCR was performed using primers EMT-F and melAT-R.

### Purification of Recombinant CopR and EMSAs

The interaction between CopR and the upstream sequence of *CopB* was verified by electrophoretic mobility shift assay (EMSA). Primers (copR-F and copRHis_6_-R) used for amplifying the CopR gene are listed in Supplementary Table S2. A His_6_ tag was introduced at the C-terminus of CopR for nickel affinity purification. The digested PCR product was inserted into pNZ8148 at the corresponding sites to obtain the recombinant plasmid pNZCopRHis_6_. This plasmid was introduced into *L. lactis* NZ9000, and recombinant CopRHis_6_ was expressed and purified using Ni Sepharose 6 Fast Flow media (GE Healthcare, Uppsala, Sweden). Purified CopRHis_6_ protein was analyzed by SDS–PAGE, and the final protein concentration was measured using a NanoDrop 2000 microspectrophotometer (NanoDrop Technologies, Wilmington, DE, United States).

Electrophoretic mobility shift assays were performed using the Lightshift Chemiluminescent EMSA Kit (Thermo Fisher Scientific, Rockford, IL, United States). DNA probes used for EMSA were obtained by annealing complementary oligos (EMSA copB-F and -R, Supplementary Table S2), which were biotin-labeled using the Biotin 3′-End DNA Labeling Kit (Thermo Scientific). Binding reactions (20 μL) contained 1× binding buffer, 50 ng μL^-1^ Poly dl-dC, 2.5% (v/v) glycerol, 0.05% (v/v) Nonidet P-40, 5 mM MgCl_2_, 20 fmol labeled probe, and 2.5 μg CopRHis_6_. In order to verify specific binding between protein and DNA, a 200-fold molar excess of unlabeled probe competitor (4 pmol; Supplementary Table S2) was added to the EMSA reaction mixture. In addition, another EMSAs were further performed under the different concentrations of Cu^2+^ (5, 10, 25, and 50 μM). Subsequent steps were carried out following the manufacturer’s instructions in both two assays.

### Copper Tolerance Assays

Starter cultures of CAUH2ΔcopR, CAUH2ΔcopB, and CAUH2ΔmelA were grown overnight at 37°C in MRS medium and inoculated into 10 mL of fresh MRS culture medium containing 1.5 mM CuSO_4_ at 37°C for 6 h. The OD_600_ was determined at 60-min intervals. All results were obtained by at least three independent experiments and each biological replicate was performed in replicates.

### Statistical Analysis

Means and standard deviations were calculated. The Student’s *t*-test was performed to investigate statistical differences. Differences between samples with *p*-values < 0.05 were considered statistically significant.

## Results

### Homologous Overexpression of the Predicted TF in *L. plantarum* CAUH2

**FIGURE 1:**
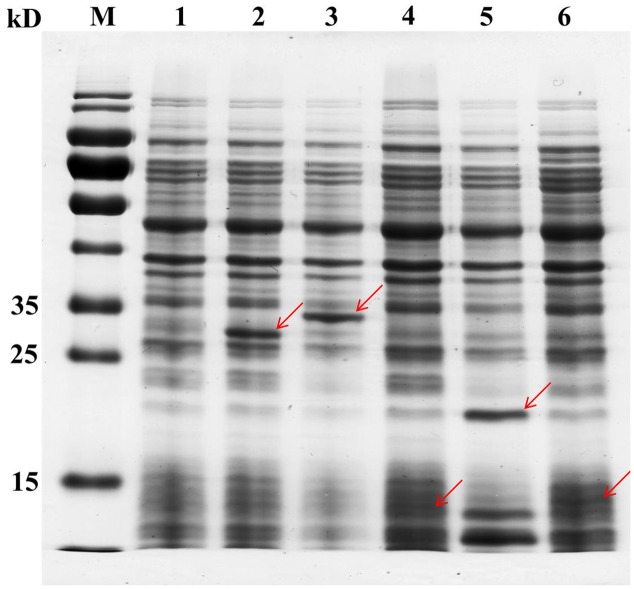
Analysis of TF overexpression in five randomly selected recombinant strains by SDS–PAGE. Cells were grown to the early logarithmic phase (OD_600_ ∼0.8), soluble extracts were boiled in 5× sample buffer, and analyzed by 12% denaturing SDS–PAGE. Lane 1, CAUH2-pSlp8148; lane 2, CAUH2-TF04; lane 3, CAUH2-TF17; lane 4, CAUH2-TF21; lane 5, CAUH2-TF32; lane 6, CAUH2-TF36. Red arrows indicate the proteins overexpressed in each sample.

Although 45 TFs were detected in the genome of CAUH2, only 43 TF genes were successfully inserted into the vector pslpA8148. Recombinant plasmids were sequenced and verified by aligning with the database genome sequence of CAUH2. The correct plasmids were designated pSlpA-TF01 to pSlpA-TF43. These plasmids were transformed into CAUH2 to generate recombination strains, designated CAUH2-TF01 to TF43. SDS–PAGE analysis of cultures from five randomly selected strains revealed the overproduction of 29, 34, 14, 21, and 15 kD proteins (**Figure 1**), which corresponded to the expected sizes of TreR1, GntR1, GlnR, FlrR, and CopR, respectively. These results confirmed the successful overexpression of the five TFs in *L. plantarum* CAUH2. We therefore extrapolated that the other TF genes would also be successfully overexpressed in the host strain *L. plantarum* CAUH2.

### Overexpression of *treR*, *mntR*, and *copR* Increased the Sensitivity of CAUH2 to H_2_O_2_

**FIGURE 2:**
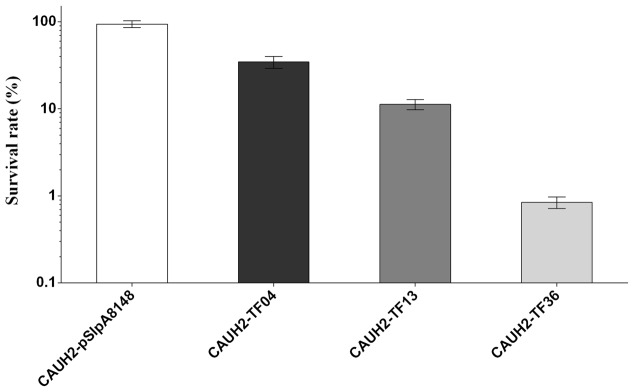
Sensitivity of partial TF overexpression strains to 5 mM H_2_O_2_. Survival rate was calculated as the ratio of the number of colonies obtained on MRS plates after and before H_2_O_2_ challenge. Data are averages from at least three independent experiments, and error bars indicate SD.

To probe the possible roles of the overexpressed TFs in oxidative stress in CAUH2, survival experiments of all 43 recombination strains were performed exposured to 5 mM H_2_O_2_. The results showed that the survival rates of strains CAUH2-TF04, CAUH2-TF13, and CAUH2-TF36 were 3-, 10-, and 110-fold lower than that of the control strain, which harbored TFs TreR1, MntR, and CopR, respectively (*p* < 0.05; **Figure 2**). These three TFs were involved in trehalose utilization, manganese homeostasis, and copper homeostasis. However, survivals of the other 40 recombinant strains were not significantly different from the control strain. These results indicate that MntR, TreR, and CopR might play an important role in H_2_O_2_ tolerance in CAUH2. Overexpression of copR caused the largest decrease in survival of the 43 TFs, and the mechanism of this gene in oxidative stress was therefore further investigated in this study.

### Insertional Inactivation of *copR* Enhances Resistance to H_2_O_2_

**FIGURE 3:**
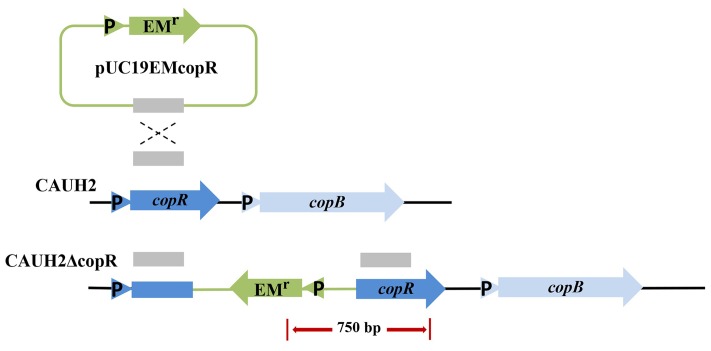
Construction of the *L. plantarum* mutant strain CAUH2ΔcopR. Genes are represented by arrows, promoters are indicated by triangles, and the internal fragment of *copR* is represented by a solid red box. Chromosomal DNA is represented by black lines, plasmid DNA is represented by blue lines, and the red arrow indicates the PCR products amplified using the forward primer EMT-F and the reverse primer copRT-R.

**FIGURE 4:**
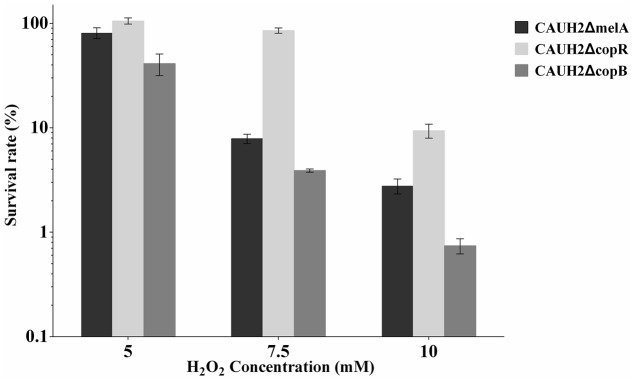
Survivals of the CAUH2ΔcopR and CAUH2ΔcopB mutants following 5, 7.5, and 10 mM H_2_O_2_ challenge. Survival rates were calculated as the ratio of the number of colonies obtained on MRS plates after and before H_2_O_2_ challenge. Data are averages from three independent experiments, and error bars indicate SD.

The *copR* mutant was constructed as shown in **Figure 3**. When chromosomal DNA from the CAUH2ΔcopR mutant was used as template for PCR, the expected 750 kb product was obtained, and sequencing revealed amplification of the expected fragments of the Em_r_ and *copR* genes, confirming the correct integration of Em_r_ into the chromosome of CAUH2 by a single crossover homologous recombination event. To eliminate the growing effects of Em selective pressure between *copR* mutant and wild strain, a *melA* gene mutant CAUH2ΔmelA was constructed as the control strain. The *melA* gene (A1F92_14365) encoding the α-galactosidase predominantly hydrolyzes the galactooligosaccharides. Thus, the inactivation of *melA* has no significant effects on CAUH2 cells growth in MRS media with glucose as the sole carbon source, which was also confirmed by the growth experiment (**Supplementary Figure S1**). Challenge assays demonstrated that the *copR* mutant showed significantly higher H_2_O_2_ resistance than the control strain, i.e., 1.3-, 10.8-, and 3.4-fold increase in survival when exposed to 5, 7.5, and 10 mM H_2_O_2_, respectively (*p* < 0.05; **Figure 4**). These results further confirmed that CopR is involved in H_2_O_2_ resistance in *L. plantarum* CAUH2.

### *In Silico* Analysis of the *copR* Gene and Its Flanking DNA Sequences

**FIGURE 5:**
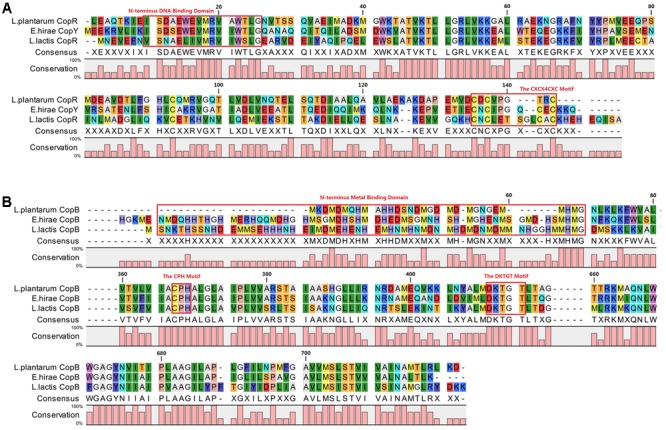
Alignment of CopR *and* CopB in *L. plantarum* CAUH2, *E. hirae*, and *L. lactis* IL1403. Alignments were obtained using ClustalX 2.1 (Larkin et al., 2007) with default settings and visualized in CLC Sequence Viewer 7.8.1. **(A)** Alignment of CopR repressors and **(B)** alignment of CopB proteins.

Amino acid sequence alignments showed that CopR of CAUH2 shares 46 and 30% identities with *E. hirae* CopY (GenBank accession no. CAA86835.1) and *L. lactis* CopR (GenBank accession no. NP_266988), respectively. The N-terminus sequences for putative DNA binding (Magnani et al., 2008) and the C-terminus CXCX_6_CXC motif for putative copper binding (Strausak and Solioz, 1997) were conserved in these three CopY-type proteins (**Figure 5A**). According to genome sequence, the putative CopR-related genes in *L. plantarum* CAUH2 show a different organization from either *E. hirae* or *L. lactis*. Analysis of the regions flanking *copR* in CAUH2 genome revealed two divergent genes: *A1F92_RS13925* encoding a hypothetical protein, and *copB* encoding a copper-translocating P-type ATPase. Moreover, homologs of the *copA* and *copZ* genes were also detected in the CAUH2 genome. These two genes are located 27 kb downstream of *copB* and form a polycistron. The promoter regions of *copR*, *copB*, *A1F92_RS13925*, and *copZ* were further searched, the *cop* box consensus sequence TACAnnTGTA was only detected in the *copB* promoter region. These results indicated that transcription of *copB* is under the control of CopR in CAUH2.

Further alignments indicated that CopB from *L. plantarum* shares 54 and 49% amino acid sequence identity with CopB from *E. hirae* and CopB from *L. lactis*. The CPH motif for putative transmembrane metal binding site and DKTGT motif for a phosphorylation domain were conserved in these three CopB proteins (Odermatt et al., 1993; **Figure 5B**). In addition, only one conserved histidine- and methionine-rich domain for putative metal binding was observed in N-terminus of CopB from CAUH2.

### CopR Binds to *cop* Box of *copB* Promoter

**FIGURE 6:**
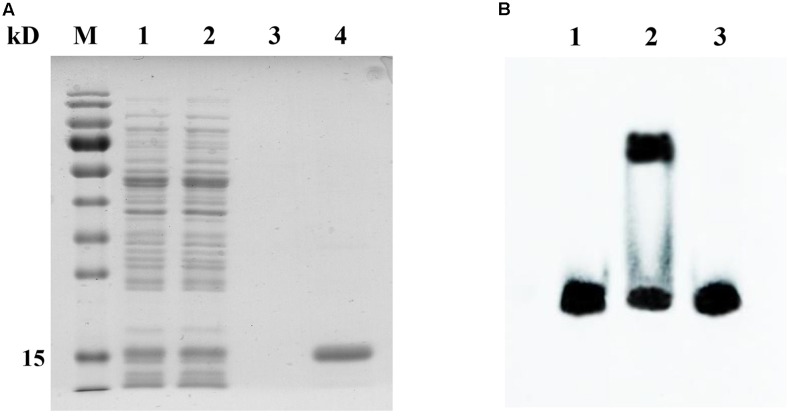
SDS–PAGE analysis of the purified CopRHis_6_ and specific binding of CopRHis_6_ to the predicted binding sites upstream of the *copB* gene. **(A)** SDS–PAGE analysis of the purified CopRHis_6_. Lanes 1 and 2, *L. lactis* NZCK and NZCopRHis_6_ with 10 ng ml^-1^ nisin induction; lanes 3 and 4, purified products of NZCK and NZCopRHis_6_. **(B)** EMSAs showing the specific interaction of CopR and the predicted binding sites upstream of *copB*. Binding reactions consisted of the following: lane 1, 20 fmol labeled probe alone; lane 2, 20 fmol labeled probe, and 2.5 μg CopRHis_6_; lane 3, 20 fmol labeled probe, 4 pmol unlabeled probe, and 2.5 μg CopRHis_6_.

Since the *copB* gene was identified as a putative target of CopR, protein–DNA interactions were analyzed by EMSA. CopR with a C-terminal His tag was expressed in *L. lactis* NZ9000 and purified by affinity chromatography. SDS–PAGE revealed a single protein band with a molecular mass of about 15 kDa, suggesting the recombinant CopRHis_6_ was successfully expressed and purified (**Figure 6A**). The EMSA results indicated that the purified CopRHis_6_ bound to biotin-labeled *copB* probes, and retarded their mobility (**Figure 6B**). Assays were further performed using unlabeled probes as specific competitors. The specific shift was abolished, which indicated specific binding of CopRHis_6_ to the *copB* probe. These results indicated that CopR specifically binds to the predicted binding sites upstream of *copB* and thereby regulates *copB* transcription.

### Insertional Inactivation of *copB* Increases the Sensitivity of CAUH2 to H_2_O_2_

In order to investigate the role of CopB in H_2_O_2_ stress, a *copB* mutant, designated CAUH2ΔcopB, was constructed by homologous recombination and subjected to challenge assays. When exposed to 5, 7.5, and 10 mM H_2_O_2_, the survival rate of CAUH2ΔcopB was 2.0-, 2.0-, and 4.0-fold lower than that of the control strain, respectively (*p* < 0.05; **Figure 4**). These results demonstrated that CopB plays a critical role in resistance to H_2_O_2_-induced oxidative stress in CAUH2.

### CopB Functions as a Cu^2+^ Induced Copper Export ATPase in CAUH2

**FIGURE 7:**
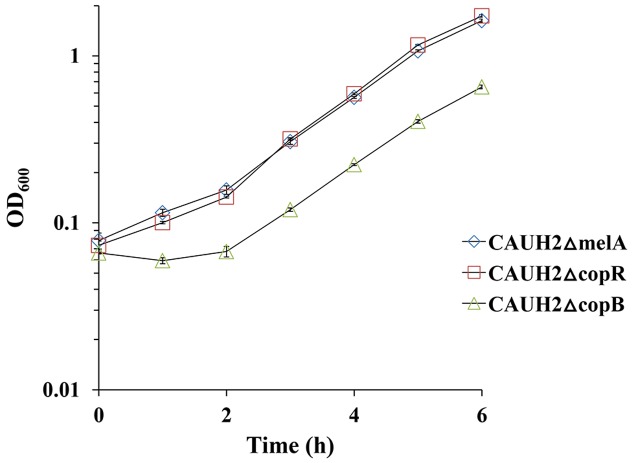
Growth of the CAUH2ΔcopB mutant in MRS containing 1.5 mM CuSO_4_. All experiments included three biologically independent replicates. The results are means of biological triplicate samples.

Given that copper can produce hydroxyl radicals from H_2_O_2_, we speculated that CopB may be involved in resistance by exporting Cu^2+^ ions from cells. To confirm whether CopB participates in the protection of CAUH2 cells as a copper ATPase, CAUH2ΔcopB cells were grown in MRS media containing 1.5 mM Cu^2+^ for 6 h. The growth was clearly retarded compared to the control strain, and significantly less biomass was achieved (**Figure 7**). These results suggest that CopB improves oxidative tolerance by exporting copper ions as an ATPase in *L. plantarum* CAUH2.

**FIGURE 8:**
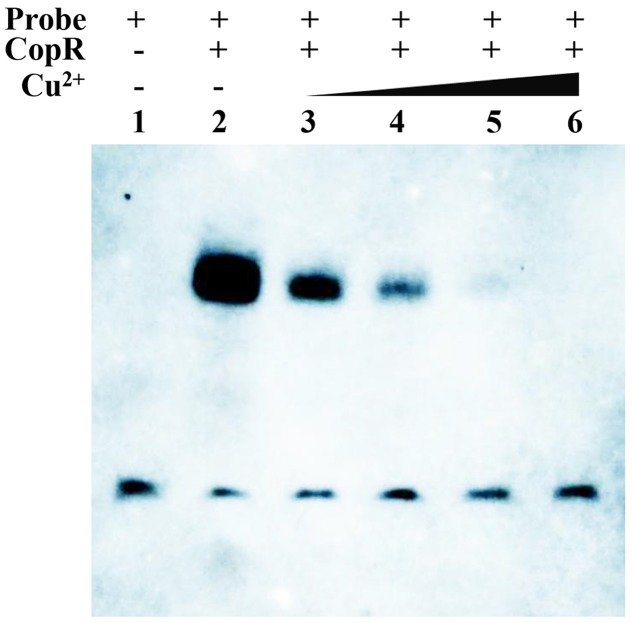
EMSAs showing the interaction of CopR and the predicted binding sites upstream of *copB* in the presence of different concentrations of Cu^2+^. Binding reactions consisted of the following: lane 1, 20 fmol labeled probe alone; lane 2, 20 fmol labeled probe and 2.5 μg CopRHis_6_; lane 3, 20 fmol labeled probe and 2.5 μg CopRHis_6_ and 5 μM Cu^2+^; lane 4, 20 fmol labeled probe, 2.5 μg CopRHis_6_, and 10 μM Cu^2+^; lane 5, 20 fmol labeled probe, 2.5 μg CopRHis_6_, and 20 μM Cu^2+^; and lane 6, 20 fmol labeled probe, 2.5 μg CopRHis_6_, and 50 μM Cu^2+^.

Since sequence alignment of CopR revealed a potential copper binding site, it was speculated that Cu binding may affect its ability to bind *cop* boxes. EMSA analysis showed that the binding intensity of CopR with the probes became weak with an increasing concentration of copper, suggesting that the CopR can be released from the *cop* box in the promoter of CopB by Cu^2+^ (**Figure 8**). These results revealed that CopR functions as a regulator that negatively regulates *copB* gene and Cu^2+^ serves as inducer of CopR to activate the ATPase CopB to export cupric ions under H_2_O_2_ stress in *L. plantarum* CAUH2.

## Discussion

The two oxidation states of copper, Cu^+^ and Cu^2+^, allow its participation in essential redox reactions, but can also promote the formation of ROS that causes cellular damage (Karlin, 1993). Thus, copper homeostasis has to be tightly regulated to preclude toxic effects in cells (Vulpe and Packman, 1995; Linder and Hazegh-Azam, 1996). Previous studies demonstrated that Cu^+^ deficiency can lead to a decrease in enzyme activity for heme-dependent catalase and glutathione peroxidase in various tissues (Chen et al., 1994; Strain, 1994). Based on the results of our transcriptome analysis on the H_2_O_2_ stress response of *L. plantarum* CAUH2 (NCBI GEO Series accession no. GSE99096), the genes encoding heme-dependent catalase (A1F92_RS14705) and glutathione peroxidase (A1F92_RS00895) were up-regulated 2.27- and 6.23-fold, respectively, suggesting CAUH2 employs these two enzymes as primary intracellular H_2_O_2_ scavengers (Abriouel et al., 2004). To maintain the activity of these enzymes in the presence of H_2_O_2_, CAUH2 cells must increase copper uptake. In *E. hirae*, the extracellular reductase CorA supplies Cu^+^ for uptake by CopA (Solioz and Stoyanov, 2003). Correspondingly, the *ndh2* gene encoding a membrane-anchored type-2 NADH dehydrogenase (A1F92_RS04560) that reduces Cu^2+^ to Cu^+^ was also detected in the CAUH2 genome (Rodriguez-Montelongo et al., 2006). Transcriptome analysis showed that the *ndh2* gene and the *copA* gene were up-regulated 4- and 14-fold under H_2_O_2_ stress, respectively, which indicates an increased requirement for copper ions in CAUH2 to maintain the activity of catalase and glutathione peroxidase under H_2_O_2_ stress.

Intracellular copper ions are generally bound to proteins as a cofactor, but they can be released to catalyze the formation of highly reactive hydroxyl radicals. In CAUH2, a copper chaperone protein (A1F92_RS14195) maybe involved in intracellular copper homeostasis as a Cu storage protein (Coyle et al., 2002; Tapia et al., 2004). A previous study found that the total Cu binding capacity of this chaperone protein was decreased when it was oxidized (Fabisiak et al., 1999). Under oxidative stress conditions, free copper ions likely accumulate in CAUH2 and damage cellular components through the formation of deleterious hydroxyl radicals. Up-regulation of CopB could enhance oxidative resistance by lowering the intracellular concentration of copper ions and preventing the damaging Fenton reaction.

**FIGURE 9:**
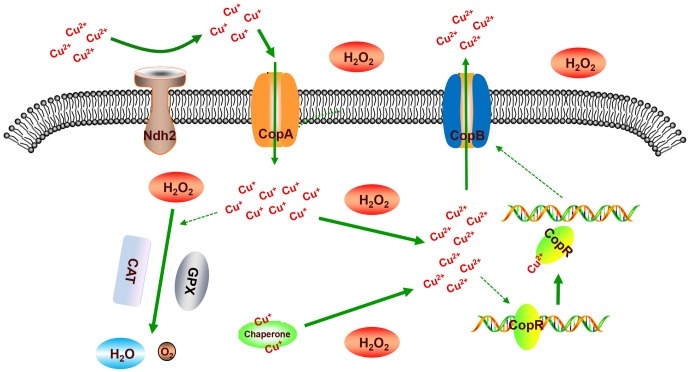
Illustration of the mechanism of CopR involved in oxidative response to H_2_O_2_ in *L. plantarum* CAUH2. “CAT” represents catalase, “GPX” represents glutathione peroxidase, “Chaperone” represents the copper chaperone protein encoded by gene *A1F92_RS14195*.

In order to clarify the mechanism of CopR involved in H_2_O_2_ stress response of *L. plantarum*, the regulation network of CopR was summarized in **Figure 9**. Under the H_2_O_2_ stress, CAUH2 cells enhance the uptaking of cuprous ions by the up-regulated expression of genes *ndh2* and *copA* to maintain the activity of heme-dependent catalase and glutathione peroxidase, which detoxify the cells by transforming H_2_O_2_ into H_2_O and O_2_. Meanwhile, both the free cuprous ions and the cuprous ions binding to copper chaperones are oxidized into cupric ions by the intracellular H_2_O_2_. As an inducer, the accumulated Cu^2+^ binds to CopR to release it from the *cop* box in *copB* promoter. Then the accumulated CopB enhances the exporting of Cu^2+^ for avoiding damage caused by copper ions. Taken altogether, our results revealed the role of copper ATPase CopB and its regulator CopR in H_2_O_2_ tolerance, which is of great importance for exploring novel H_2_O_2_-detoxifying mechanisms in *L. plantarum* and other bacteria.

## Author Contributions

YH and YY designed the study. YY, JY, JL, QX, and TL performed the experiments. YY analyzed and evaluated the data. YY and YH wrote the manuscript. YH revised the manuscript. All authors read and approved the final manuscript.

## Conflict of Interest Statement

The authors declare that the research was conducted in the absence of any commercial or financial relationships that could be construed as a potential conflict of interest.

## Footnotes

1 http://regprecise.lbl.gov/RegPrecise/propagated_genome.jsp?pgp_collection_id=11&genome_id=224

## Supplementary Material

FIGURE S1 Growth of wild-type strain CAUH2 and the mutants CAUH2ΔcopR, CAUH2ΔcopB, and CAUH2ΔmelA. CAUH2 was grown in MRS media, CAUH2ΔcopR, CAUH2ΔcopB, and CAUH2ΔmelA were grown in MRS media supplemented with 10 μg mL^-1^ erythromycin.
