# Supplementary figures and images for: The Copper Homeostasis Transcription Factor CopR Is Involved in H2O2 Stress in Lactobacillus plantarum CAUH2
Source: Front Microbiol. 2017 Oct 17;8:2015. doi: 10.3389/fmicb.2017.02015 (PMC5651008; doi:10.3389/fmicb.2017.02015)

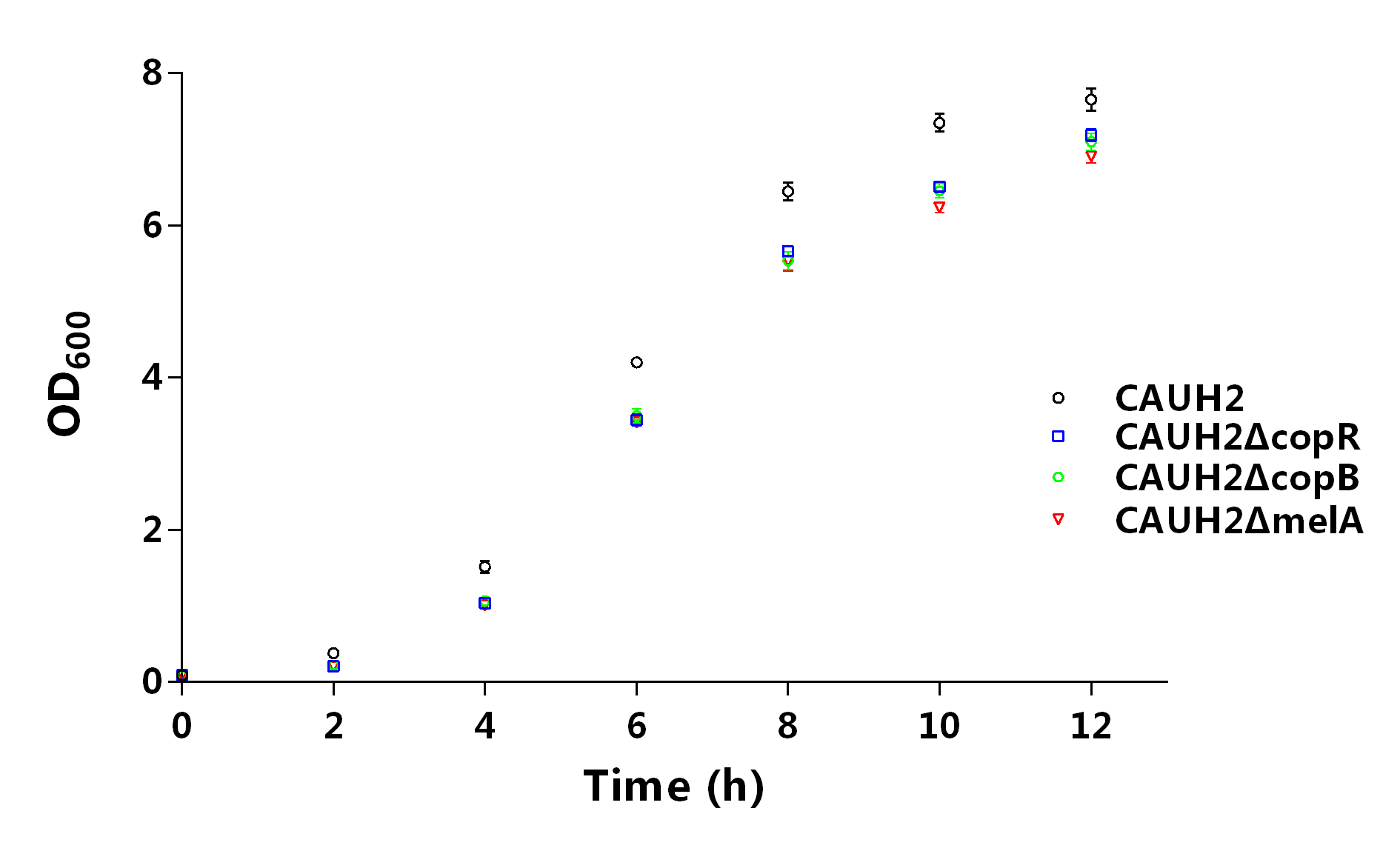

Supplement: FIGURE S1 — Growth of wild-type strain CAUH2 and the mutants CAUH2ΔcopR, CAUH2ΔcopB, and CAUH2ΔmelA. CAUH2 was grown in MRS media, CAUH2ΔcopR, CAUH2ΔcopB, and CAUH2ΔmelA were grown in MRS media supplemented with 10 μg mL-1 erythromycin. [file Image_1.JPEG]
