# Supplementary material for: The Copper Homeostasis Transcription Factor CopR Is Involved in H2O2 Stress in Lactobacillus plantarum CAUH2
Source: Front Microbiol. 2017 Oct 17;8:2015. doi: 10.3389/fmicb.2017.02015 (PMC5651008; doi:10.3389/fmicb.2017.02015)
Supplement: Supplementary file 2 [file Table_1.DOCX]

Supplementary Material

The copper homeostasis transcription factor CopR is involved in H_2_O_2_ stress in *Lactobacillus plantarum* CAUH2

Yang Yang^1^, Jia Yin^1^, Jie Liu^1^, Tian Lan^1^, Fazheng Ren^1,2^, Yanling Hao^1,2*^

*** Correspondence: Yanling Hao: haoyl@cau.edu.cn**

# Supplementary Tables

**Table S1.** Homologues of TF genes present in *Lactobaicllus plantarum* CAUH2 with the corresponding overexpression strains and primers.

| Gene ID | Gene | Description | Predicted molecular weight (Da) | Overexpression plasmid | Overexpression strain | Primers: Sequence 5’–3’ |
| --- | --- | --- | --- | --- | --- | --- |
| A1F92_RS00695 | *mdxR* | Maltose/Maltodextrin utilization | 36,191 | pSlpA-TF01 | CAUH2-TF01 | mdxR-F: CATGCCATGGGAATAACGCTAAACGATGT |
|  |  |  |  |  |  | mdxR-R: CCCAAGCTTTTAACGGGTCGATTCAGC |
| A1F92_RS00765 | *scrR* | Sucrose utilization | 36,231 | pSlpA-TF01 | CAUH2-TF02 | scrR-F: CGGGGTACCATGAAACCAAAATTAAA |
|  |  |  |  |  |  | scrR-R: CCCAAGCTTTTACGCGGTAAGGCCC |
| A1F92_RS00935 | *mtlR* | Mannitol utilization | 78,021 | pSlpA-TF03 | CAUH2-TF03 | mtlR-F: CATGCCATGGGCAAAGTACGTAGAA |
|  |  |  |  |  |  | mtlR-R: CCCAAGCTTCTATTTCGTTAGTTCGTTC |
| A1F92_RS01065 | *treR1* | Trehalose utilization | 26,899 | pSlpA-TF04 | CAUH2-TF04 | treR1-F: CATGCCATGGTGAATAAAGCGGCC |
|  |  |  |  |  |  | treR1-R: CCCAAGCTTTTATTTCTTAGTTCGCCGAGC |
| A1F92_RS01355 | *ytrA* | Hypothetical ABC transporter | 13,272 | pSlpA-TF05 | CAUH2- TF05 | ytrA-F: CATGCCATGGGCCAAATTGATCAT |
|  |  |  |  |  |  | ytrA-R: CCCAAGCTTTTATTTTTCCCCCATTTG |
| A1F92_RS01415 | *birA1* | Biotin biosynthesis | 35,318 | pSlpA-TF06 | CAUH2- TF06 | birA1-F: CATGCCATGGGCACAACTAAAGAACAAGTC |
|  |  |  |  |  |  | birA1-R: CCCAAGCTTTCATACTGGTAACCCTACCCTG |
| A1F92_RS01925 | *purR* | Purine metabolism | 30,564 | pSlpA-TF07 | CAUH2- TF07 | purR1-F: CATGCCATGGGCAAAGATCGTAGAA |
|  |  |  |  |  |  | purR1-R: CCCAAGCTTCTAGACTTCCGCACCGA |
| A1F92_RS02320 | *nagR* | N-acetylglucosamine utilization | 26,334 | pSlpA-TF08 | CAUH2- TF08 | nagR-F: CATGCCATGGGTAGTTCGCCAA |
|  |  |  |  |  |  | nagR-R: CCCAAGCTTTTATTTTTCAAGATAAAACTCAA |
| A1F92_ RS03030 | *rex* | Energy metabolism | 25,214 | pSlpA-TF09 | CAUH2- TF09 | rex-F: CATGCCATGGGCGCAGAAACAAAAATTC |
|  |  |  |  |  |  | rex-R: CCCAAGCTTTTAATCTTCAGTCTCATT |
| A1F92_ RS03325 | *cggR* | Glycolysis | 38,076 | pSlpA-TF10 | CAUH2- TF10 | cggR-F: CATGCCATGGGCGGCAGAAACAAAAATTC |
|  |  |  |  |  |  | cggR-R: CGCAAGCTTTTATTTCTTAGTTCGCCGAGC |
| A1F92_ RS03615 | *birA2* | Biotin biosynthesis | 35,408 | pSlpA-TF11 | CAUH2- TF11 | birA2-F: CGGGGTACCATGCATTCAGATATTC |
|  |  |  |  |  |  | birA2-R: CCCAAGCTTCTAGCCATGATAACCACCG |
| A1F92_ RS03665 | *perR1* | Manganese homeostasis; Iron homeostasis; | 16,896 | pSlpA-TF12 | CAUH2- TF12 | perR1-F: CATGCCATGGGTGAAACACCAATTGCCC |
|  |  |  |  |  |  | perR1-R: CCCAAGCTTTCAGCTATTAATTGCCTGCTT |
| A1F92_ RS04260 | *mntR* | Manganese homeostasis | 23,935 | pSlpA-TF13 | CAUH2- TF13 | mntR-F: CATGCCATGGGCACCCCCATGA |
|  |  |  |  |  |  | mntR-R: CCCAAGCTTTTACTTTACAAAGACATTGTG |
| A1F92_ RS04365 | *ctsR* | Heat shock response | 17,614 | pSlpA-TF14 | CAUH2- TF14 | ctsR-F: CATGCCATGGGCCAAAGTCAAAATAT |
|  |  |  |  |  |  | ctsR-R: CCCAAGCTTTTAGCTTTCGTAACGCA |
| A1F92_ RS04750 | *mleR1* | Malate utilization | 32,918 | pSlpA-TF15 | CAUH2- TF15 | mleR1-F: CATGCCATGGGTAATACACGTGATTTAG |
|  |  |  |  |  |  | mleR1-R: CCCAAGCTTTTAAATCTTCATTTTAAG |
| A1F92_ RS04760 | *mleR2* | Malate utilization | 34,330 | pSlpA-TF16 | CAUH2- TF16 | mleR2-F: CATGCCATGGGCAACACAAAAGACT |
|  |  |  |  |  |  | mleR2-R: CCCAAGCTTTTAATCGGTCTGATCAGTG |
| A1F92_ RS04915 | *gntR1* | Gluconate utilization | 31,726 | pSlpA-TF17 | CAUH2- TF17 | gntR1-F: CATGCCATGGGATCCACACAATCAC |
|  |  |  |  |  |  | gntR1-R: TGCTCTAGATTCAAGCTCCGTTCGTGTT |
| A1F92_ RS06030 | *argR1* | Arginine degradation/biosynthesis | 17,126 | pSlpA-TF18 | CAUH2- TF18 | argR1-F: CATGCCATGGGTAAAAAGT |
|  |  |  |  |  |  | argR1-R: CTAGTCTAGATCAGTCTTCATTACTA |
| A1F92_ RS06170 | *flpA1* | Heavy metal resistance | 23,985 | pSlpA-TF19 | CAUH2- TF19 | flpA1-F: CATGCCATGGGACATTCAAAAATGGAT |
|  |  |  |  |  |  | flpA1-R: GGGGAGCTCCTAACAAATTGTAATCTG |
| A1F92_ RS06330 | *nrdR* | Deoxyribonucleotide biosynthesis | 19,886 | pSlpA-TF20 | CAUH2- TF20 | nrdR-F: CATGCCATGGGTCAATGTCCACA |
|  |  |  |  |  |  | nrdR-R: CCCAAGCTTCTAATCTTTTTTTCGTTTAGG |
| A1F92_ RS06610 | *glnR* | Nitrogen assimilation | 14,552 | pSlpA-TF21 | CAUH2- TF21 | glnR-F: CATGCCATGGGAAAGGAAAAGGAAC |
|  |  |  |  |  |  | glnR-F: CCCAAGCTTTTAGTGTGCCGGATAAT |
| A1F92_ RS06715 | *argR2* | Arginine degradation/biosynthesis | 17,452 | pSlpA-TF22 | CAUH2- TF22 | argR2-F: CATGCCATGGTGAAGAAGCAAGAGC |
|  |  |  |  |  |  | argR2-R: CCCAAGCTTTTAGTGATCACTCAGTAAGCG |
| A1F92_ RS08190 | *ytrA* | Hypothetical ABC transporter | 14,436 | pSlpA-TF23 | CAUH2- TF23 | ytrA-F: CATGCCATGGGTCCGTTTGC |
|  |  |  |  |  |  | ytrA-R: CCCAAGCTTTTAACTTGGATACTCATCCCG |
| A1F92_ RS08395 | *hrcA* | Heat shock response | 38,909 | pSlpA-TF24 | CAUH2- TF24 | hrcA-F: CATGCCATGGGCATCACGTTAACTG |
|  |  |  |  |  |  | hrcA-R: CCCAAGCTTCTACTTGGAACTTGCTCGCTC |
| A1F92_ RS08555 | *lexA* | SOS response | 23,302 | pSlpA-TF25 | CAUH2- TF25 | lexA-F: CATGCCATGGGAAGTAAAACATCGGA |
|  |  |  |  |  |  | lexA-R: CCCAAGCTTTTACTGAAACAGCATGTC |
| A1F92_ RS08685 | *fruR* | Fructose utilization | 27,699 | pSlpA-TF26 | CAUH2- TF26 | furR-F: CATGCCATGGGTGCTTACAGAAG |
|  |  |  |  |  |  | furR-R: TGCTCTAGATCATGCTTGAACCTCCT |
| A1F92_ RS09500 | *ccpA* | Carbon catabolism | 36,330 | pSlpA-TF27 | CAUH2- TF27 | ccpA-F: CATGCCATGGGAGAAAAACAAACAGTAACA |
|  |  |  |  |  |  | ccpA-R: CCCAAGCTTTTAATCAGCAGACTTGGTTGA |
| A1F92_ RS10540 | *nrtR* | NAD biosynthesis | 28,067 | Unsuccessful | Unsuccessful | nrtR-F: CTAGCCATGGGTGCGCATGAAGTCA |
|  |  |  |  |  |  | nrtR-R: CCCAAGCTTCTACTTTAGCCGGTAT |
| A1F92_ RS10835 | *pflR* | Formate metabolism | 28,305 | pSlpA-TF28 | CAUH2- TF28 | pflR-F: CGGGGTACCATGAATCAACGCAA |
|  |  |  |  |  |  | pflR-R: CTAGTCTAGATTAATGAGTCGCAACAGTC |
| A1F92_ RS10860 | *ccpB* | Carbon catabolism | 34,923 | pSlpA-TF29 | CAUH2- TF29 | ccpB-F: CATGCCATGGGAACCAATATCCATGACATT |
|  |  |  |  |  |  | ccpB-R: CCCAAGCTTCTATCTTTCAATAAATTCTGATT |
| A1F92_ RS11390 | *mdeR* | Multidrug efflux; Multidrug resistance | 14,081 | pSlpA-TF30 | CAUH2- TF30 | mdeR-F: CATGCCATGGGCCAGTTCAACTTTAATAGC |
|  |  |  |  |  |  | mdeR-R: CCCAAGCTTTCATGATTCGGAAAACCC |
| A1F92_ RS11515 | *niaR* | NAD biosynthesis | 18,701 | pSlpA-TF31 | CAUH2- TF31 | niaR-F: CATGCCATGGGAACAGGATCAGAACG |
|  |  |  |  |  |  | niaR-R: CCCAAGCTTTTAATTTTAATACAAATAACC |
| A1F92_ RS12175 | *treR2* | Trehalose utilization | 27,260 | Unsuccessful | Unsuccessful | treR2-F: CATGCCATGGGAGGGAAACAAACA |
|  |  |  |  |  |  | treR2-R: CCCAAGCTTTTAATTACGAATTAATTC |
| A1F92_ RS12855 | *flrR* | Drug resistance | 21,319 | pSlpA-TF32 | CAUH2- TF32 | flrR-F: CATGCCATGGGAGCAGCGACCAAT |
|  |  |  |  |  |  | flrR-R: CCCAAGCTTTCATTTTTGAGCCTGAGT |
| A1F92_ RS13335 | *ywzG* | Transport | 12,063 | pSlpA-TF33 | CAUH2- TF33 | ywzG-F: CATGCCATGGGAGCCATTCAAATTAG |
|  |  |  |  |  |  | ywzG-R: CCCAAGCTTTCATGATTCATCCTCCC |
| A1F92_RS13360 |  | Sugar utilization | 36,742 | pSlpA-TF34 | CAUH2- TF34 | 13360-F: CATGCCATGGGAACAACGATTAG |
|  |  |  |  |  |  | 13360-R: TGCTCTAGATTAAGCACTTTTTCTTTTG |
| A1F92_ RS13475 | *perR2* | Manganese homeostasis; Iron homeostasis | 18,130 | pSlpA-TF35 | CAUH2- TF35 | perR2-F: CATGCCATGGTGGCAGAAGATATG |
|  |  |  |  |  |  | perR2-R: CCCAAGCTTCTATTCACTGTGCGTC |
| A1F92_ RS13920 | *copR* | Copper homeostasis | 15,682 | pSlpA-TF36 | CAUH2- TF36 | copR-F: CATGCCATGGGCTTGGAAGCACAAACT |
|  |  |  |  |  |  | copR-R: CCCAAGCTTTTAACACCGCGTCCCC |
| A1F92_ RS14200 | *flpA2* | Heavy metal resistance | 24,528 | pSlpA-TF37 | CAUH2- TF37 | flpA2-F: CATGCCATGGGAGCAGAACATGAATGTGTGC |
|  |  |  |  |  |  | flpA2-R: GGGGAGCTCTTATTCAGTTAATAATAGCTG |
| A1F92_ RS14295 | *lacR* | Lactose utilization | 9,879 | pSlpA-TF38 | CAUH2- TF38 | lacR-F: CATGCCATGGTGCCGACTATTCGTG |
|  |  |  |  |  |  | lacR-R: CCCAAGCTTCTATGATTTATTCGTCGG |
| A1F92_ RS14335 | *galR1* | Galactosides/Galactose utilization | 38,222 | pSlpA-TF39 | CAUH2- TF39 | galR1-F: CATGCCATGGGTACGATGGCCGT |
|  |  |  |  |  |  | galR1-R: TGCTCTAGATCATTTGGTGCTTTCACG |
| A1F92_ RS14380 | *galR2* | Galactosides/Galactose utilization | 37,105 | pSlpA-TF40 | CAUH2- TF40 | galR2-F: CATGCCATGGGTGCGGCGACGCTAAAAG |
|  |  |  |  |  |  | galR2-R: CCCAAGCTTCTACTTGGAACTTGCTCGCTC |
| A1F92_ RS14630 | *araR* | Arabinose utilization | 40,930 | pSlpA-TF41 | CAUH2- TF41 | araR-F: CATGCCATGGGTGAAAATAAATATC |
|  |  |  |  |  |  | araR-R: CTAGTCTAGATTAATTTAAGTCTGCT |
| A1F92_ RS15055 | *rbsR* | Ribose utilization | 36,855 | pSlpA-TF42 | CAUH2- TF42 | rbsR-F: CGGGGTACCATGAAAAATAAGGCAG |
|  |  |  |  |  |  | rbsR-R: CCCAAGCTTTTAATTCAAATGTGCCG |
| A1F92_ RS15070 | *padR* | Phenolic acid stress response | 21,080 | pSlpA-TF43 | CAUH2- TF43 | PadR-F: CATGCCATGGGAGCGCAAAAAAACA |
|  |  |  |  |  |  | PadR-R: CCCAAGCTTTTACTCCGGTAAGGCTTTC |

Restriction enzyme cutting sites are underlined: CCATGG-*Nco*I, AAGCTT-*Hin*dIII, GGTACC-*Kpn*I, TCTAGA-*Xba*I,.

**Table S2.** Oligonucleotides and primers used in this study

| **Oligonucleotides/ primers** | **Sequence (5’-3’)** |
| --- | --- |
| EM-F | AACTGCAGTTCAAATATGTATCCGC |
| EM-R | CCCAAGCTTTTACCAATGCTTAATC |
| copRHA-F | CTAGTCTAGAATCAGTGATGCCGAAT |
| copRHA-R | CCGGAATTCGTTTGGTTAACAAGGTC |
| copBHA-F | CTAGTCTAGAGCAGGTCCATGACGTGG |
| copBHA-R | CCGGAATTCTGATAGTGCTAACGCC |
| melAHA-F | CTAGTCTAGACGAAATGTTTGTGCTGGAT |
| melAHA-R | CCGGAATTCAATCGGGTCATTATTATCTG |
| EMT-F | TTTTAGCAAACCCGTATTCCAC |
| copRT-R | ACACAATCGCAGTC CACCAT |
| melAT-R | ACACAATCGCAGTCCACCAT |
| copR-F | CATGCCATGGGCTTGGAAGCACAAACT |
| copRHis_6_-R | CCCAAGCTTAATGATGATGATGATGATGACACCGCGTCCCC |
| EMSA copB-F | GTGTTTTTCTTGACTTTTATCGTC**TACAATTGTA**AACTTTAGCCATAAAT**TACAGATGTA**GACAAT |
| EMSA copB-R | ATTGTCTACATCTGTAATTTATGGCTAAAGTTTACAATTGTAGACGATAAAAGTCAAGAAAAACAC |
| Unlabeled competitor **-**F | ATTGTC**TACAATTGTA**AACTTTAGCCATAAAT**TACAGATGTA**GACGA |
| Unlabeled competitor **-**R | TCGTCTACATCTGTAATTTATGGCTAAAGTTTACAATTGTAGACAAT |

Restriction enzyme cutting sites are underlined: CTGCAG-*Pst*I, AAGCTT-*Hin*dIII, GAATTC-*Eco*RI, GGTACC-*Kpn*I, TCTAGA-*Xba*I, CCATGG-*Nco*I.

Boldfaces indicate the predicted binding sites of CopR.
